# Cannabinoid Modulation of the Stressed Hippocampus

**DOI:** 10.3389/fnmol.2017.00411

**Published:** 2017-12-19

**Authors:** Franciele F. Scarante, Carla Vila-Verde, Vinícius L. Detoni, Nilson C. Ferreira-Junior, Francisco S. Guimarães, Alline C. Campos

**Affiliations:** Department of Pharmacology, School of Medicine of Ribeirão Preto, Centre for Interdisciplinary Research on Applied Neurosciences (NAPNA), Cannabinoid Research Institute, University of São Paulo, São Paulo, Brazil

**Keywords:** hippocampus, stress, HPA axis, endocannabinoid system, CB_1_, CB_2_, neuroplasticity

## Abstract

Exposure to stressful situations is one of the risk factors for the precipitation of several psychiatric disorders, including Major Depressive Disorder, Posttraumatic Stress Disorder and Schizophrenia. The hippocampal formation is a forebrain structure highly associated with emotional, learning and memory processes; being particularly vulnerable to stress. Exposure to stressful stimuli leads to neuroplastic changes and imbalance between inhibitory/excitatory networks. These changes have been associated with an impaired hippocampal function. Endocannabinoids (eCB) are one of the main systems controlling both excitatory and inhibitory neurotransmission, as well as neuroplasticity within the hippocampus. Cannabinoids receptors are highly expressed in the hippocampus, and several lines of evidence suggest that facilitation of cannabinoid signaling within this brain region prevents stress-induced behavioral changes. Also, chronic stress modulates hippocampal CB_1_ receptors expression and endocannabinoid levels. Moreover, cannabinoids participate in mechanisms related to synaptic plasticity and adult neurogenesis. Here, we discussed the main findings supporting the involvement of hippocampal cannabinoid neurotransmission in stress-induced behavioral and neuroplastic changes.

## Cannabinoid Signaling

**Figure 1:**
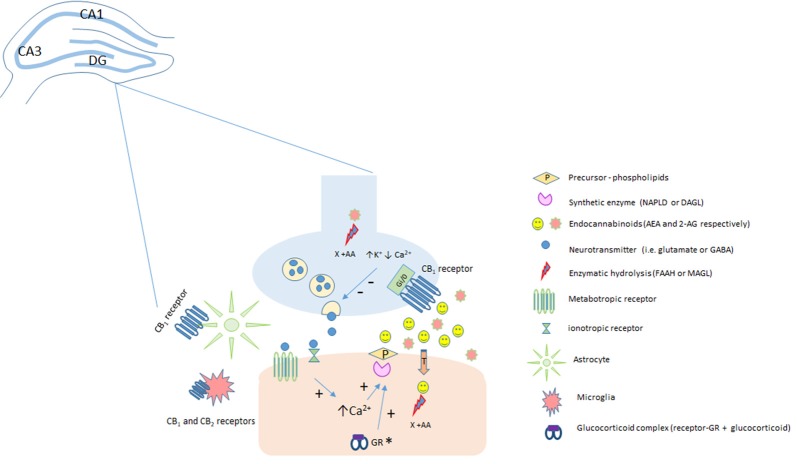
Classical representation of endocannabinoid modulation in the hippocampus. Anandamide (AEA) and 2-AG are produced “on demand” in a calcium (Ca^2+^)-dependent manner (via the previous activation of a metabotropic or ionotropic receptor in the post-synaptic terminal). After the synthesis of endocannabinoids (eCBs) by specialized enzymes, they can act as retrograde messengers by activating CB_1_ receptors located at pre-synaptic terminals. In neurons, CB_1_ is a Gi/o-coupled receptor, and its activation reduces Ca2+ currents and increases K+ currents, leading to the inhibition of neurotransmitter release. Fatty-acid amide hydrolase (FAAH) and monoacylglycerol lipase (MAGL) enzymes hydrolyze AEA (postsynaptically) and 2-AG (presynaptically), respectively, limiting eCB action. The CB_1_ receptor is also expressed in astrocytes and microglia and the CB_2_ receptor is expressed in activated microglia and putatively expressed in neurons (still under debate). *It has been speculated, that in the hippocampus, stress-induced activation of the HPA could lead, depending on genomic actions of glucocorticoids acting at glucocorticoid receptors, to an increase in 2-AG levels. 2-AG, 2-arachidonoylglycerol; AEA, anandamide; CB_1_, type 1 cannabinoid receptor; CB_2_, type 2 cannabinoid receptor; DAGL, diacylglycerol lipase; FAAH, fatty acid amide hydrolase; GR, glucocorticoid receptors; MAGL, monoacylglycerol lipase; NAPE-PLD, N-acyl phosphatidylethanolamine-specific phospholipase D.

Humankind has been using* Cannabis sativa* for therapeutic and recreational purposes since ancient times (Mechoulam and Parker, 2013). In the early 60s, Raphael Mechoulam’s group isolated and described the chemical structures of more than 60 compounds, named cannabinoids, present in this plant (Gaoni and Mechoulam, 1964). Following this achievement, it was demonstrated in the 80s that the effects of cannabimimetic drugs were mediated by their interaction with specific sites, resulting in the activation of a G protein signaling and the inhibition of adenylate cyclase activity (Howlett and Fleming, 1984; Howlett, 1985). Devane et al. (1988) identified a specific binding site for Δ9-tetrahydrocannabinol (THC; the main psychotomimetic compound of the plant) in the rat brain. Later, this binding site was cloned and named Cannabinoid type 1 receptor or CB_1_ (Matsuda et al., 1990). A few years later, a second cannabinoid receptor, CB_2_, was also described (Munro et al., 1993). Together, these findings provided the basis for the elucidation of a complete new endogenous system: the endocannabinoid system (eCBS). In parallel with the discovery of the two-cannabinoid receptors, endogenous ligands, named endocannabinoids (eCB), were also described (Devane et al., 1992; Mechoulam et al., 1995). So far, the most studied eCB are the ones derived from membrane phospholipids, particularly arachidonoyl ethanolamide or anandamide (AEA), and 2-arachidonoyl glycerol (2-AG; Maccarrone et al., 2014). eCB are recognized now as neuromodulators synthetized “on demand” after cell depolarization or receptor stimulation (e.g., NMDA, mGlu5) by specific enzymes (AEA: N-acyl-phosphatidylethanolamine phospholipase, D-NAPE-PLD; 2-AG: α and β isoforms of diacylglycerol lipase, DAGL; Saito et al., 2010). Fatty-acid amide hydrolase (FAAH) and monoacylglycerol lipase (MAGL) are the main enzymes that metabolize AEA and 2-AG, respectively, ending eCB actions (Cravatt et al., 1996; Dinh et al., 2004; Figure 1).

CB_1_ receptors are widely distributed in the Central Nervous System (CNS) and primarily expressed in pre-synaptic terminals where eCBs can act as retrograde messengers (Wilson and Nicoll, 2001; Maccarrone et al., 2014). In the CNS, this G_i/0_-coupled protein receptor is densely expressed in neurons. Once activated, it leads to a decreased probability of neurotransmitter release (GABA, glutamate, etc.), via inhibition of presynaptic Ca^2+^ channels and activation of K^+^ channels (Mackie et al., 1995; Kreitzer et al., 2001).

eCBs are involved in short- and long-term plasticity in several brain structures such as the amygdala (Azad et al., 2004), nucleus accumbens (Robbe et al., 2002), striatum (Gerdeman et al., 2002) and hippocampus (Ohno-Shosaku et al., 2007; Zhu and Lovinger, 2007; Izumi and Zorumski, 2012). Despite the canonical signaling pathways via G_i/0_-coupled protein receptors, studies indicate the existence of CB_1_ receptors coupled to G_q_ proteins in hippocampal neurons (Lauckner et al., 2005) and astrocytes (Navarrete and Araque, 2010; Lutz et al., 2016). eCBs released by neurons can bind to Gq-coupled CB_1_ receptors present in astrocytes, thus promoting glutamate release from these glial cells (Navarrete and Araque, 2010; Lutz et al., 2016).

Although still under debate, some studies have also suggested the neuronal expression of CB_2_ receptors (Brusco et al., 2008; Li and Kim, 2016; Stempel et al., 2016). However, others report that the messenger RNA expression of this cannabinoid receptor is negligible (400–2000-fold times lower than CB_1_ receptor mRNA) in the CNS (Marco et al., 2014). CB_2_ is expressed in primed microglial cells (Núñez et al., 2004), influencing the microglia phenotypes by polarizing these cells to M2-type (alternative/anti-inflammatory; Mecha et al., 2015; Li and Kim, 2017) through the cAMP/PKA pathway (Tao et al., 2016).

The precise endogenous function of eCBs is still under investigation, but several lines of evidence suggest that impairment of eCB neuromodulation is associated with neuropsychiatric disorders such as major depressive disorders (MD), schizophrenia and anxiety disorders (Hillard et al., 2012; Pacher and Kunos, 2013). Supporting this proposal, for example, rimonabant, a CB_1_ receptor antagonist/inverse agonist, which years ago was marketed for the treatment of tobacco abuse, obesity and associated metabolic disorders, was withdrawn from the market due to its psychiatric side effects (Moreira and Crippa, 2009). Major depressive patients had a significantly higher frequency of a mutant allele of the CNR1 (rs1049353; Monteleone et al., 2010). Accordingly, CB_1_ receptor had a higher expression in the prefrontal cortex of patients with major depressive disorder (Choi et al., 2012). Recently, it was also observed a significant increase in CB_1_ expression, and a reduction in mechanisms of DNA methylation at the promoter of CNR1, the gene coding for the CB_1_ receptor in schizophrenia patients (D’Addario et al., 2017). The CNR1 gene is located at the chromosome 6q14-15, a locus possible related to susceptibility for bipolar affective disorder (Rice et al., 1997; Abou Jamra et al., 2010). Moreover, a direct association between CNR1, CNR2 and FAAH polymorphisms and bipolar disorder susceptibility has also been described (Monteleone et al., 2010; Minocci et al., 2011).

Exposure to stressful situations changes eCB signaling and increases the susceptibility to psychiatric disorders (Hill et al., 2010; Hillard, 2014). Cannabinoid receptors, eCBs (AEA and 2-AG), and the enzymes responsible for their synthesis and deactivation are widely expressed in the hippocampus (Tsou et al., 1998; Katona et al., 1999). Impairments in hippocampus-dependent functions (e.g., cognitive deficits, affect lability and dysregulated pattern separation) are a common feature of psychiatric patients (David et al., 2010; Christian et al., 2014; Kang et al., 2016). Therefore, in the next topic of the present manuscript, we revised studies that have explored the role of hippocampal eCB-signaling during stressful situations.

## Stress Modulation of eCB Signaling in the Hippocampus

**Figure 2:**
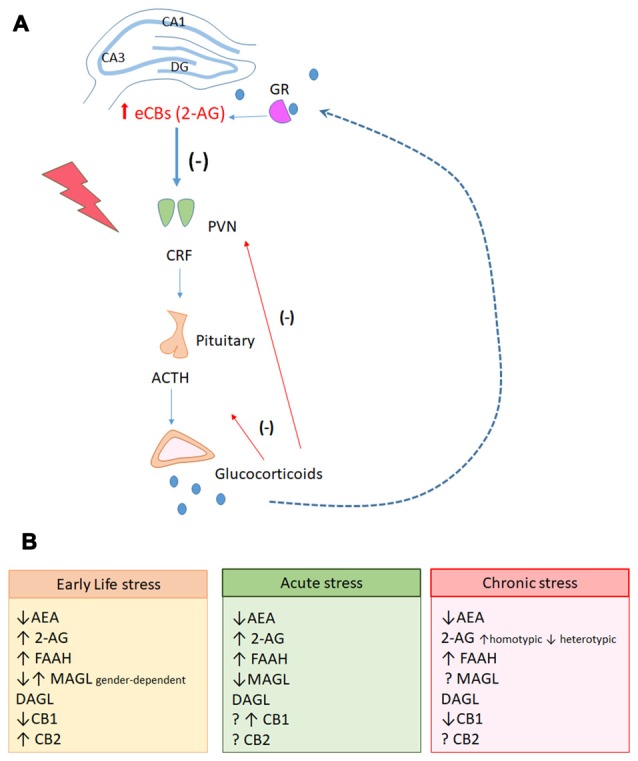
**(A)** Hippocampal endocannabinoid-mediated signaling participates in the negative feedback of the Hypothalamic-pituitary-adrenal (HPA) axis. During exposure to stressful events, neurons located in the paraventricular nuclei of hypothalamus (PVN) actively secrete corticotrophin-released-hormone (CHR) in the pituitary portal system. In the pituitary, the secretion of the adrenocorticotropic hormone (ACTH) is stimulated. Circulating ACTH reaches the cortex of the adrenal gland and stimulates the production and secretion of glucocorticoids. The PVN receives projections from limbic areas, including the hippocampus that participates in the glucocorticoid-mediated negative feedback inhibition of the HPA axis. It has been hypothesized that during stress response, activation of glucocorticoid receptors in the hippocampus could induce the production of 2-AG that would activate CB_1_ receptors in GABAergic interneurons, leading to disinhibition of glutamatergic neurons. These latter neurons would send projections to GABAergic neurons in the PVN, inhibiting corticotrophin release hormone (CRH) release and stopping the stress response. **(B)** Stress responses have complex modulation on the genes related to the endocannabinoid system (eCBS) in the hippocampus, depending on individual factors (gender, age) and on the intensity, duration and nature of the stress.

Several studies strongly suggest that eCBs reduce hypothalamic–pituitary–adrenal (HPA) axis activation and facilitate appropriate stress recovery (Patel et al., 2004, 2009; Balsevich et al., 2017). However, the impact of stressful experiences in the eCBs appears to be quite complex, depending on the intensity, duration, and nature/type of the stressor, and the brain region investigated (Patel et al., 2004; Rubino et al., 2008; Campos et al., 2010; Hill et al., 2010; Figure 2).

The hippocampus is a forebrain structure that presents a very high expression of CB_1_ receptors (Tsou et al., 1998), mainly in cholecystokinin (CCK)-positive GABAergic interneurons (Katona et al., 1999). In addition, lower expression of CB_1_ receptors can be also found in glutamatergic, serotonergic, and cholinergic neurons. Also present in glial cells, CB_1_ receptors participate in the neuron-astrocyte communication in the hippocampus (Navarrete and Araque, 2008).

The hippocampal formation has been recognized as an important site for the deleterious effects of uncontrollable stress in brain function (McEwen, 1999). The hippocampus negatively modulates the HPA axis, one of the main players in stress regulation in mammals. HPA activation leads to the release of glucocorticoids, mediators of the countless effects of stress on the hippocampus function (Kim et al., 1996).

Pharmacological and genetic studies support the role of eCBs as regulators of emotional processing in the hippocampus (Akirav, 2011; Jenniches et al., 2016). However, conflicting results have been observed, probably because of the diverse components of the eCB system, the wide influence of CB_1_ receptor signaling in different neuronal circuits, and external factors, such as stress protocols, animal strain, treatment dosage, and even the choice of the pharmacological/genetic tool (Hill and Gorzalka, 2005; Roohbakhsh et al., 2007; Dubreucq et al., 2012; Reich et al., 2013; Korem and Akirav, 2014; Zhong et al., 2014; Zhang et al., 2015).

### Nature, Duration and Controllability of Stress and eCBS

AEA and 2-AG could differently modulate emotional-related responses. In brain areas related to neuropsychiatric disorders, exposure to acute stress leads to a rapid decrease of AEA while inducing a delayed increased in 2-AG levels (Hill and Tasker, 2012). In the hippocampus, exposure to restraint (Wang et al., 2012) or social defeat stress (Dubreucq et al., 2012) reduces the levels of AEA whereas increases 2-AG. Interestingly, early life stressors (maternal separation and prenatal restraint stress) also decreased AEA levels in the hippocampus but increased 2-AG in male rodents (Llorente et al., 2008; Marco et al., 2013). Stress-induced changes in eCBs levels could be partially mediated by increased FAAH activity (Navarria et al., 2014) and reduction of 2-AG hydrolysis by MAGL (Suárez et al., 2010). The stress-effect on MAGL, however, has generated conflicting results and appears to be gender-dependent (Marco et al., 2014). On the other hand, 2-AG content seems to be influenced by corticosterone levels in acute stress protocols (Hill et al., 2011b; Atsak et al., 2012; Wang et al., 2012).

The severity and the nature of the stress protocol could differently modulate eCBs. In foot-shock based protocols, changes in AEA content seem to depend on the intensity and duration of the foot-shock protocol, as well as the time-window of the analysis. For example, Morena et al. (2014) described increased AEA levels 10 min after the stress in limbic areas, including the hippocampus, of rodents submitted to 0.45-mA/1 s foot-shock protocol. In contrast, another study, using six foot-shocks of 0.7 mA, found a reduction in AEA levels in the hippocampus 24 h after the end of the stress protocol (Bluett et al., 2014). In these two studies, however, no changes in 2-AG levels were observed in the hippocampus (Bluett et al., 2014; Morena et al., 2014).

Even if there are contradictory results (Bortolato et al., 2007; Lomazzo et al., 2015), the effect of homotypic chronic stress on eCB levels are similar to those observed after acute stress. They include an early decreased of AEA levels (due to increased FAAH activity) and a delayed increase in 2-AG levels (due to reduced MAGL activity; Hill et al., 2010; Dubreucq et al., 2012). Repeated restraint stress elevated 2-AG levels exclusively in the amygdala, while social defeat stress increased 2-AG content only in the hippocampus (Hill et al., 2010; Dubreucq et al., 2012). Some studies in rodents submitted to heterotypic protocols, such as the chronic unpredictable stress (CUS), report reduced AEA and 2-AG levels (Hill et al., 2008b; Zhong et al., 2014), and increased FAAH expression in the hippocampus (Reich et al., 2009), while others show no changes in eCBs levels after CUS protocols (Bortolato et al., 2007).

Stress can also influence CB_1_ function. Korem and Akirav (2014) demonstrated that a single footshock episode increased CB_1_ receptors expression in the CA_1_ region of the hippocampus (Korem and Akirav, 2014). On the other hand, chronic stress is associated with a down-regulation of CB_1_ receptor signaling in this brain structure (Hill and Gorzalka, 2005; Hill et al., 2008b; Reich et al., 2009; Hu et al., 2011; Lee and Hill, 2013). Stress-induced CB_1_-downregulation in the hippocampus appears to involve corticosterone. The repeated administration of this glucocorticoid mimics the effects of chronic stress on CB_1_ receptor expression and function (Hill et al., 2008a; Bowles et al., 2012). A similar panorama is observed in rodents exposed to early-life stressful events (Suárez et al., 2009).

CB_2_ receptors are also modified by stress exposure. For example, early maternal deprivation increases CB_2_ immunoreactivity in the rat hippocampus (Suárez et al., 2009). A recent study verified that high-levels of anxiety, when combined with aversive factors (fear conditioning or exercise), increases CB_2_ receptor gene expression in the hippocampus. The opposite is also true: decreased anxiety (measured by escape response) was associated with reduced CB_2_ gene expression (Robertson et al., 2017). In mice, overexpressing CB_2_ receptors decreases anxiety-like behaviors, which is partially explained by changes in hippocampal GABA_A_ receptors (García-Gutiérrez and Manzanares, 2011).

### Interplay between Glucocorticoids and Cannabinoid Neurotransmission

Considering that stress-induced HPA axis activation and consequent corticosterone release modulate the production of eCB and CB_1_ function (Hillard et al., 2016), it is possible that cannabinoids and glucocorticoids interact to control stress responses. Glucocorticoid hormones and their receptors (mineralocorticoid-MR and glucocorticoid receptors-GR) are abundantly expressed in the hippocampus (Herman et al., 1993), where, together with eCBs, they control the negative feedback regulation of the HPA axis (Hillard et al., 2016). Several pieces of evidence indicate that glucocorticoids and the eCB system interact to control emotional, physiological, and adaptive responses to stress. However, the precise mechanisms involved in the cross-talk between these systems within the hippocampus are not entirely understood (Hill and Tasker, 2012).

Stress and glucocorticoids induce endocannabinoid synthesis, which in turn can act to limit HPA axis-induced changes in the brain fear circuitry (amygdala–hippocampal–cortico–striatal circuit; Steiner and Wotjak, 2008; de Oliveira Alvares et al., 2010; Hill et al., 2011b; Wang et al., 2012). The basolateral nucleus of the amygdaloidal complex (BLA) participates in the regulation of the HPA axis. Interesting, in the BLA, AEA contributes to eCB tonic signaling, which is disrupted in rodents submitted to stress-based protocols presenting high serum levels of corticosterone (Ganon-Elazar and Akirav, 2009; Hill et al., 2009, 2011b; Wang et al., 2012).

A stressful situation or corticosterone administration cause endocannabinoid-mediated suppression of GABAergic transmission in the hippocampus (Wang et al., 2012). In this forebrain structure, activation of GR receptors facilitates 2-AG-mediated neuromodulation. After repeated presentation of chronic homotypic stressors (e.g., chronic restraint stress), 2-AG levels progressively increased in the hippocampus (Hill and Tasker, 2012). Thus, it is reasonable to suggest that, in this condition, 2-AG contributes to stress adaptations induced by HPA axis activation (Hill et al., 2010; Dubreucq et al., 2012). In the prefrontal cortex, 2-AG-mediated activation of CB_1_ receptors located in GABAergic terminals facilitates HPA axis negative feedback (Hill et al., 2011b; Radley and Sawchenko, 2011; Wang et al., 2012). Decreased GABA release induced by CB_1_ stimulation in this brain area would facilitate the activation of cortical glutamatergic neurons. These latter neurons would signal to GABAergic interneurons located in the bed nucleus of the stria terminalis (BNST), which ultimately inhibit the secretion of the corticotrophin release hormone (CRH) in the PVN. Although not yet investigated, it is possible that a similar phenomenon occurs in the hippocampus, since a significant part of CB_1_ receptors expressed there are located in GABAergic terminals (Tsou et al., 1998).

On the other hand, some studies suggest a possible biphasic effect of glucocorticoid signaling in the eCB system (Hill et al., 2008a; Wang et al., 2012; Reich et al., 2013). Administration of WIN55, 212-2 (non-selective cannabinoid agonist) prevented the trauma-induced behavioral impairments and normalized the expression of CB_1_ and GRs receptors in the hippocampus (CA_1_ subfield; Korem and Akirav, 2014). Likewise, the same group found that intrahippocampal (into the ventral subiculum area) and intra-BLA injections of WIN55, 212-2 prevented stress-induced impairments in fear extinction retention, an effect blocked by a GRs antagonist. These results reinforce the notion that eCBs and glucocorticoids interact in these two brain structures during stressful situations (Ganon-Elazar and Akirav, 2013). The balance between these two systems, therefore, could be crucial for stress-coping mechanisms. Corroborating this proposal, Eldridge and Landfield (1990) reported that adrenalectomized rats treated for 14 days with THC, a partial agonist of CB_1_ receptors, presented down-regulation of hippocampal GR binding, a similar result induced by the administration of high doses of glucocorticoid.

## Stress Induces Changes in Cannabinoid-Mediated Hippocampal Neuroplasticity

Probably reflecting its role in several brain functions such as learning, memory and affective processing, the hippocampus is a very plastic brain structure. One of the primary mechanisms of synaptic plasticity, particularly relevant to learning and memory, is the phenomena of long-term potentiation (LTP; Morris et al., 1986). It was first identified in the perforant pathway of the hippocampus (Lomo, 1966), and was further characterized in the Shaffer collaterals and mossy fibers. This process, therefore, affects the synaptic strength of the tri-synaptic circuit (Alger and Teyler, 1976). Another process that modulates synaptic efficiency, the long-term depression (LTD), was characterized in hippocampal synapses shortly after the description of LTP (Lynch et al., 1977; Dunwiddie and Lynch, 1978). The changes in the hippocampal synaptic efficiency are accompanied by modifications in dendritic arbor and dendritic spines (Woolley et al., 1990; Fuchs et al., 1995; Magariños and McEwen, 1995; Engert and Bonhoeffer, 1999; Toni et al., 1999; Frick et al., 2004; Matsuzaki et al., 2004; Mehta, 2004; Monfils et al., 2004; Chen et al., 2008).

Changes in hippocampal-dependent functions (e.g., cognitive deficits, affect lability and dysregulated pattern separation) are common symptoms in psychiatric patients. They are also a frequent phenotype observed in animal models of psychiatric disorders, including major depressive and anxiety disorders, schizophrenia and addiction (David et al., 2010; Christian et al., 2014; Kang et al., 2016). These symptoms could be indicative of a disrupted neuroplasticity mechanisms, such as adult newly generated neurons and atrophy of dendritic arbor of the mature neurons (Yun et al., 2016). Indeed, a decrease in hippocampal size has been considered a cellular substrate of major depression (Sheline, 1996; Duman et al., 2000; Kempermann and Kronenberg, 2003), posttraumatic stress disorder (Kitayama et al., 2005; Karl et al., 2006) and schizophrenia (Goldman and Mitchell, 2004).

The integrity of the machinery responsible for neuroplasticity seems crucial for the proper acquisition of stress coping responses (Snyder et al., 2011; Canal et al., 2014; Sinha et al., 2016; for review, see Levone et al., 2015; McEwen et al., 2015). Stress exposure, however, can also induce maladaptive changes that might contribute to the negative outcomes of chronic stress, such as learning and memory deficits, anxiogenesis, and changes in stress-coping behaviors (Willner et al., 1992; D’Aquila et al., 1994; Luine et al., 1994). For example, Foy et al. (1987) demonstrated that stress impairs hippocampal LTP induction in the CA_1_ region, whereas Watanabe et al. (1992) showed that repeated restraint stress for 21 days reduces dendritic branching of CA3 pyramidal neurons. Additionally, the neuroplastic hypothesis of depression and the mechanisms antidepressant drugs is heavily supported by studies using chronic stress. Selective serotonin reuptake inhibitors or tricyclic antidepressants reverse or prevent stress neuroplastic outcomes such as decreased adult hippocampal neurogenesis, dendritic arborization and spine density, and deficits in BDNF production in hippocampal and cortical neurons (Smith et al., 1995; Magariños et al., 1996; Czéh et al., 2002; Pham et al., 2003). The time-course of these antidepressant neuroplastic consequences correlates well with the latency needed for the therapeutical effects of these drugs (Santarelli et al., 2003; Bessa et al., 2008).

Adult hippocampal neurogenesis describes a stress-vulnerable process (Gould et al., 1997; Czéh et al., 2002; Pham et al., 2003; Heine et al., 2004) that gives birth to new granular cells. These cell are incorporated into the local circuitry, connecting the dentate gyrus to the CA3 region through the mossy fibers (Kempermann et al., 2015). This process has been implicated in learning and memory (Gould et al., 1999; for a review, see Kempermann, 2002), and behaviors related to anxiety and depression (Sapolsky, 2004; Revest et al., 2009). Attenuation of hippocampal neurogenesis induces anxiety- and behavior- despair in rodents (Liu et al., 2008; Revest et al., 2009; Jenniches et al., 2016). Adult hippocampal neurogenesis has been suggested to buffer the stress response (Snyder et al., 2011; Campos et al., 2013) and is implicated in the therapeutic effect of antidepressants (Santarelli et al., 2003; Malberg, 2004; David et al., 2009). Of note, structural changes observed in the hippocampus after stress response can be attenuated or reversed by antidepressants, atypical antipsychotics and physical exercise, which are known to positively impact hippocampal neurogenesis (Kempermann et al., 2010; Erickson et al., 2011).

The role of the eCBS in the fine-tuning of excitatory and inhibitory neurotransmissions makes it a very relevant modulator of neuroplasticity (den Boon et al., 2015; Song et al., 2015). In the 1970s, some lines of evidence began to suggest that THC could interfere in the release of neurotransmitters such as GABA, serotonin, noradrenaline and excitatory amino acids (Ho et al., 1971; Sklenovský et al., 1974; Banerjee et al., 1975; Mahfouz et al., 1975). Nowicky et al. (1987), showed that THC has a biphasic effect in the duration of LTP in rat hippocampal slices, with lower concentrations increasing and higher concentrations decreasing the duration of LTP. During the following years, the existence of the eCBS was revealed to the world, and several studies started investigating its role in neuroplastic responses. Terranova et al. (1995) showed that AEA inhibits the induction of LTP in the CA_1_ region, an effect abolished by a CB_1_ receptor antagonist. Stella et al. (1997) evidenced a similar CB_1_-dependent effect of 2-AG. Additionally, Paton et al. (1998) and Misner and Sullivan (1999) showed that cannabinoid receptor activation resulted, respectively, in a higher paired-pulse depression and reduced frequency, but not amplitude, of miniature excitatory post-synaptic currents in CA_1_ neurons. Altogether, these results indicated that the cannabinoid receptor activation directly interferes with the pre-synaptic release of neurotransmitters. These discoveries contributed to the attribution of two phenomena of short-term synaptic plasticity, depolarization-induced suppression of inhibition (DSI) and depolarization-induced suppression of excitation (DSE), to the retrograde signaling mediated by eCBs (Kreitzer and Regehr, 2001; Wilson and Nicoll, 2001; Wilson et al., 2001).

A form of LTD is also dependent on endocannabinoid signaling, as evidenced by Marsicano et al. (2002) in the amygdala, and by Chevaleyre and Castillo (2003) in hippocampal synapses. This effect is dependent on the cAMP/PKA pathway (Chevaleyre et al., 2007). Additionally, Chávez et al. (2010) have shown that AEA induces LTD in the dentate gyrus in a TRPV1 receptor-dependent manner. Wang W. et al. (2017) showed that the activation of CB_1_ receptors modulates the synaptic responses of hippocampal pathways in different ways. In the Schaffer Commissural pathway, CB_1_ activation decreased excitatory post-synaptic potential and impaired LTP induction via an ERK1/2-MUNC18-1-dependent neurotransmitter release. In the lateral perforant pathway, however, CB_1_ activation leads to a FAK/ROCK2-dependent signaling that, through a β-integrin cooperative action, affects the actin dynamics needed to stabilize the synaptic potentiation. Besides depending on the signaling pathway activated, the cannabinoid-mediated plasticity is also time-dependent. For example, sub-threshold dosages of CB_1_ agonists, that acutely do not affect, increase the phosphorylation of the transcription factor cAMP response element binding-protein (CREB) after chronic administration (Isokawa, 2009).

CB_2_ receptor deletion in mice reduced dendritic spines, impaired LTP and decreased excitatory neurotransmission (Kim and Li, 2015; Li and Kim, 2016). On the other hand, CB_1_ knock-out (KO) mice presented a depressive-like phenotype, which was accompanied by decreased BDNF levels in the hippocampus (Aso et al., 2008). Jin et al. (2004) also showed that hippocampal neurogenesis was impaired in CB_1_ KO mice, data corroborated by Aguado et al. (2005, 2007). Wolf et al. (2010) further supported the involvement of eCBS in regulating neurogenesis by showing that running and environmental enrichment, two pro-neurogenic stimuli, increase the hippocampal levels of the CB_1_ receptor. The pro-proliferative effect of running and the pro-survival effect of environmental enrichment in the neurogenic process were absent in CB_1_ KO mice. Jiang et al. (2005) showed that the cannabinoid drug HU210 exerted pro-proliferative actions through the activation of the CB_1_-ERK1/2 signaling in the dentate gyrus of the hippocampus. There is also evidence for a role of CB_2_ receptors in controlling the phenomena of adult neurogenesis (Palazuelos et al., 2006, 2012; Goncalves et al., 2008).

Cannabinoids also prevent the neuroplastic events triggered by stress. Exposure to predator odor decreased the proliferative rate of neural stem cells located in the dentate gyrus, an effect inhibited by the treatment with the endocannabinoid uptake inhibitor AM404, but not by the concomitant treatment with AM404 and the CB_1_ antagonist AM251 (Hill et al., 2006). Campos et al. (2013) found that mice exposed to CUS for 14 days presented a reduced labeling for the proliferative marker 5-bromo-2’-deoxyuridine (BrdU) and doublecortin expression, a marker of immature neurons, in the dentate gyrus, supporting an anti-neurogenic action of stress. Repeated treatment with cannabidiol, a non-psychotomimetic compound of *Cannabis sativa*, counteracted these stress outcomes.

Similarly, the MAGL inhibitor, JZL184, reversed the effects of chronic stress in adult neurogenesis and blocked the stress-induced impairment of LTP induction in the lateral perforant pathway (Zhang et al., 2015). Furthermore, Shoshan et al. (2017) evidenced that the CB_1_/CB_2_ agonist WIN55-212, 2 and the FAAH inhibitor URB597 partially blocked the impairment of LTP induction in the CA_1_ region of rats after exposure to a severe stressful event. Besides, the antidepressant-like action of the FAAH inhibitor PF3845 in mice facing an acute stress seems to be dependent on the induction of LTD in glutamatergic synapses of the Shaffer collaterals (Wang and Zhang, 2017). In addition to interfering with glutamatergic synapses, cannabinoid drugs might also affect stress responses in GABAergic and serotonergic synapses (Bambico et al., 2012, 2016; Wang Y. et al., 2017).

Concerning the signaling pathways involved in the effects of cannabinoid drugs in stress-related neuroplasticity, the mechanistic (or mammalian) target of rapamycin (mTOR) protein has been implicated in synaptogenesis, proliferative processes, and synaptic strength modulation (Li et al., 2010; Ma et al., 2010; Romine et al., 2015; Liang et al., 2016). Some studies showed that different paradigms of stress exposure reduce mTOR activity (Iiu et al., 2016; Xia et al., 2016; Zhuang et al., 2016; Seo et al., 2017), and cannabinoid drugs can interfere with this signaling pathway (Puighermanal et al., 2009; Palazuelos et al., 2012; Gobira et al., 2015; Giacoppo et al., 2017). For example, Zhong et al. (2014) demonstrated that the antidepressant-like responses trigged by JZL184, an inhibitor of 2-AG hydrolysis, in chronically stressed mice were dependent on mTOR signaling.

Stress changes endocannabinoid signaling and the modulation of neuroplastic changes. Reich et al. (2013) found that in adolescent rats exposed to chronic mild stress, CB_1_ activation facilitates CA_1_ excitatory transmission. However, in non-stressed rats, CB_1_ activation reduced the excitatory post-synaptic currents. In addition, Talani et al. (2016) mimicked the effects of food restriction on glutamate release, LTP induction, and BDNF release in the hippocampus, by blocking CB_1_ receptors. Hu et al. (2011) showed that rats exposed to chronic restraint stress presented an impaired endocannabinoid-mediated DSI in the CA_1_ pyramidal neurons of the dorsal hippocampus. Similar alterations in endocannabinoid-mediated plasticity were reported in other brain regions, such as the amygdala, habenula, striatum and the BNST (Rossi et al., 2008; Patel et al., 2009; Glangetas et al., 2013; Di et al., 2016; Park et al., 2017).

Notably, a recent work reported that chronic low doses of THC restored cognitive function in old mice by enhancing the expression of synaptic marker proteins and increasing hippocampal spine density. In old mice, THC treatment restored hippocampal gene transcription, an effect critically dependent on glutamatergic CB_1_ receptors and histone acetylation (Bilkei-Gorzo et al., 2017).

Taken together, the data presented here suggested that the eCBS buffers stress responses in the hippocampus by preventing stress-induced changes in neuroplasticity.

## Stress-Induced Modulation of the eCB Signaling in the Hippocampus: Implications for Stress Coping and Defensive Behaviors

A large number of pharmacological and genetic studies support the proposal that the eCB system is an essential regulator of cognition, mood and anxiety (for review, see Ruehle et al., 2012; Hill and Patel, 2013). Stressful experiences have been associated with the precipitation of psychiatric disorders and, as discussed above, cannabinoids play a pivotal role in the regulation of behavioral responses to stressful challenges (Patel et al., 2009). Impairment of CB_1_ signaling induces a moderate increase in anxiety-responses under basal conditions (Haller et al., 2004b). Other studies, however, using CB_1_ receptor-deficient animals or CB_1_ antagonist, revealed increased defensive responses only under high aversive conditions (Haller et al., 2004a; de Oliveira Alvares et al., 2005; Jacob et al., 2009). Under challenging environments, a significant enhancement of emotional responses induced by stress is also found after CB_1_ receptor blocked (Hill et al., 2011a; Gamble-George et al., 2013). Despite these findings, contradictory data also exist. These contradictions probably reflect the already discussed stress-dependent plasticity of the eCB system and the localization of CB_1_ receptors in distinct subpopulations of GABAergic and glutamatergic neurons. Several observations indicate that GABAergic and glutamatergic neurotransmissions have opposite actions on anxiety responses (Millan, 2003).

In the hippocampus, the effects of cannabinoids are also complex and often depend on the aversiveness of the task and the doses of drugs being tested. CB_1_ receptor agonists usually promote biphasic effects, with lower doses being anxiolytic (Zarrindast et al., 2010) while higher doses induce no effects or even anxiogenic responses (Roohbakhsh et al., 2007). In the same way, injections of THC into the ventral hippocampus or prefrontal cortex induce anxiolytic- and anxiogenic-like effects at low and high doses, respectively (Rubino et al., 2008). Laaris et al. (2010) suggest that these biphasic effects depend on THC binding to CB_1_ receptors present in GABAergic or glutamatergic synapses.

Exposure-based therapy, which relies on extinction processes after repeatedly exposing the patient to stimuli associated with a traumatic, fear-related memory, is a therapeutic approach used to treat anxiety disorders. Several studies indicate that the eCBs modulate fear memory (Marsicano et al., 2002; Morena et al., 2014), and THC prevents the recovery of fear memory (Rabinak et al., 2013). In rats submitted to contextual fear conditioning, a stress-induced learned fear paradigm, intra-dorsal hippocampus injections of the CB_1_ antagonist/inverse agonist AM251, facilitated fear expression (freezing behavior + autonomic responses, Spiacci et al., 2016). Interestingly, both the freezing behavior and autonomic responses were attenuated by previous intra-hippocampal administration of the antagonist of NMDA receptors, AP7 (Spiacci et al., 2016), suggesting that AM251 effects are being mediated by facilitation of glutamate release.

Rodents trained under a high arousal condition showed increased levels of AEA in the amygdala, medial prefrontal cortex (mPFC) and hippocampus, brain regions closely associated with fear conditioning (Morena et al., 2014). The FAAH inhibitor URB597 enhanced memory consolidation/retention when infused into these brain areas; an effect prevented by a CB_1_ antagonist (Morena et al., 2014). Microinjection of the CB_1_/CB_2_ agonist WIN55, 212-2 or an inhibitor of eCB reuptake (AM404) into the CA_1_ facilitated the extinction process of fear memory by impairing spatial learning and LTP formation. Both effects were also prevented by previous treatment with a CB_1_ receptor antagonist (Abush and Akirav, 2010). In contrast, de Oliveira Alvares et al. (2010) verified that a prior stressful experience or dexamethasone injection facilitated memory consolidation in a weak conditioning protocol, an event prevented by intrahippocampal injection of AM251. However, intrahippocampal infusion of the CB_1_ antagonist failed to abolish the disruptive effect of norepinephrine on memory retrieval (Atsak et al., 2012).

The inhibition of AEA uptake by AM404 attenuated predator scent-induced activation of defensive burying and suppression of cell proliferation in the hippocampus (Hill et al., 2006). However, the administration of AM404 into the ventral portions of the hippocampus produced opposite effects depending on previous stressful experience. Campos et al. (2010) observed that in non-stressed mice submitted to the elevated plus maze (EPM), an ethological based animal model largely used for the screening of putative anti-anxiety compounds, AM404 induces anxiogenic-like effects. However, the same dose produced anxiolytic-like effects in rats that had been previously (24 h) submitted to a 2-h protocol of restraint stress. It also decreased anxiety in the Vogel Conflict Test (VCT), a model based on punished-conflict induced by 24 h water deprivation (Campos et al., 2010). Of note, both the anxiolytic and anxiogenic responses were attenuated by AM251. It had been previously demonstrated that chronic stress impairs CB_1_ receptor-mediated short-term plasticity at GABAergic synapses in the hippocampus (Hu et al., 2011). To explain these results, the authors hypothesized that the anxiogenic-like behavior in the EPM observed in naïve rats could be due the activation the CB_1_ receptors on GABAergic terminals. The previous exposure to stressors (restraint and water deprivation) could down-regulate CB_1_ receptors located in GABAergic terminals, facilitating CB_1_-mediated attenuation of glutamate release and resulting in an anxiolytic response (Campos et al., 2010; Ruehle et al., 2012). It is also possible that stress reduces GABAergic control over hippocampal projection neurons, impairing its control over the HPA axis. However, these stress-dependent effects of AEA in the ventral hippocampus do not seem to extend to its dorsal region, since URB597 and AM404 induced anxiolytic-like effects independent of previous stress experience (Hakimizadeh et al., 2012; Lisboa et al., 2015).

Concerning 2-AG signaling, the genetic deletion of DAGL adversely affects the emotional state of animals. DAGL KO mice present a marked decreased in 2-AG levels in several brains areas, including the hippocampus, together with reduced hippocampal neurogenesis (Jenniches et al., 2016). Accordingly, inhibition of 2-AG signaling in hippocampal glutamatergic neurons by genetic overexpression of MAGL leads to decreased 2-AG levels and increased anxiety-like behavior, but no change in aversive memory formation (Guggenhuber et al., 2015).

Regarding the CB_2_ receptor, its overexpression in mice increases resistance to anxiogenic-like stimuli and modifies GABA_A_ receptors in the hippocampus and amygdala (García-Gutiérrez and Manzanares, 2011). On the other hand, acute administration of a CB_2_ antagonist induced anxiogenic effect, whereas chronic pharmacological blockade of this receptor produced anxiolytic effects associated with increased expression of the CB_2_ and the main anxiolytic subunits of GABA_A_ receptors (α_2_ and γ_2_ subunits) genes in the amygdala and the cortex. Conversely, the protein expression of these receptors was reduced in the same brain areas (García-Gutiérrez et al., 2012). The picture, however, may be more complicated, since the protein expression of these receptors was reduced in the same brain areas (García-Gutiérrez et al., 2012).

In slices of entorhinal cortex-hippocampal, Morgan et al. (2009) showed a suppression of GABAergic inhibitory signaling following the administration of the CB_2_ receptor agonist, JWH133, an effect reversed by AM630, a CB_2_ receptor inverse agonist. However, the precise mechanism involved in CB_2_-induced change in GABAergic neurotransmission remains to be fully elucidated. One possible speculation is that CB_2_ is functionally (and more) expressed in presynaptic GABAergic neurons (CB_1_- like function). Therefore, chronic administration of CB_2_ antagonist would increase the probability of GABA release. On the other hand, but in the same line of thought, after acute blocked, CB_2_ could disturb the balance between GABAergic and glutamatergic neurotransmission or induce the production of other non-classic neurotransmitters such as nitric oxide. In fact, after focal brain injury, the CB_2_ receptor agonist JWH-015 enhances the expression of the neuronal nitric oxide synthase (nNOS) enzyme (Oddi et al., 2012). In the hippocampus, inhibition of nNOS induces antidepressant-like effects (Joca and Guimarães, 2006; Hiroaki-Sato et al., 2014), and genes related to nitric oxide effects have been associated with posttraumatic stress disorder, major depressive disorder and anxiety (Bruenig et al., 2017).

On the topic of mood disorders, suppression of endocannabinoid signaling in the hippocampus is sufficient to induce a depressive-like state (Hill and Gorzalka, 2005; Hill et al., 2008b; Dubreucq et al., 2012; Valverde and Torrens, 2012). Impairment of CB_1_ receptor signaling resulted in increased immobility (passive coping behavior) in animal models used for the screening of antidepressant drugs, such as the tail suspension (TST) and the forced swimming (FST) tests. Moreover, increased HPA axis activity following exposure to stress is also reported after CB_1_ receptors signaling blocked (Aso et al., 2008). Chronic pharmacological activation of CB_1_ receptors by HU-210 induces anxiolytic and antidepressant effects and facilitates adult hippocampal neurogenesis (Jiang et al., 2005). Likewise, local activation of CB_1_ receptors within the dentate gyrus of the hippocampus elicited antidepressant-like behavior in socially isolated animals (McLaughlin et al., 2007). Of note, Wistar Kyoto (WKY) rat, a well-accepted model of depressive-like behavior, expresses high levels of CB_1_ receptors in the hippocampus. This increase of CB_1_ receptor binding sites could be a compensatory mechanism in response to diminished AEA tone in the hippocampus (Vinod et al., 2012). As with other cannabinoid effects discussed so far, there is also at least one contradictory result, with both THC and rimonabant (CB_1_ antagonist/inverse agonist) reducing immobility time in in FST after intraperitoneal injections (Häring et al., 2013).

In the case of CB_2_ receptors, few data are available, and the results are not always consistent. In an animal model based on the surgical removal of the olfactory bulbs (bulbectomy—an animal model of depression), decreased levels of AEA and CB_2_ expression in the hippocampus were reported (Smaga et al., 2017). Furthermore, overexpression of CB_2_ receptors induced a depression-resilient phenotype after chronic stress, but no alterations in hippocampal BDNF levels. However, in wild-type animals submitted to CUS, acute and chronic treatment with AM630, a CB_2_ receptor antagonist, prevented stress-induced depressive-like behaviors and decreased expression of CB_2_ and BDNF in the hippocampus (García-Gutiérrez et al., 2010).

Chronic stress produces behavioral and neurochemical changes in rodents that resemble those found in human depression. As we have already mentioned, chronic heterotypic stress (chronic mild/unpredictable/varied stress) reduces the hippocampal levels of AEA, 2-AG, CB_1_ and CB_2_ receptors (Hill and Gorzalka, 2005; Hill et al., 2008b; Reich et al., 2009; García-Gutiérrez et al., 2010; Zhong et al., 2014), and increases FAAH protein expression (Reich et al., 2009).

CUS induced depressive-like behavior, reduced hippocampal activation of mTOR signaling, decreased neurogenesis and impaired LTP induction in the hippocampus, whereas repeated injections of a MAGL inhibitor prevented CUS-induced biochemical, cellular and behavioral abnormalities (Zhong et al., 2014; Zhang et al., 2015). Recently Wang Y. et al. (2017) found that low-doses of MAGL inhibitors prevented the acute stress-induced decrease of sucrose consumption through astrocyte-mediated LTD at CA3-CA_1_ glutamatergic synapses mechanism. On the other hand, in chronically stressed mice, low doses of MAGL inhibitors induced “pro-depressive” effects whereas high-doses produced significant anti-anhedonia effects; both responses mediated by CB_1_ receptors. It is worthy to mention that, in some studies, the gene deletion or the chronic treatment with a high-dose of a MAGL inhibitor induces a pro-depressant phenotype, attributable to CB_1_ receptor downregulation (Schlosburg et al., 2010; Imperatore et al., 2015). Of note, CB_1_ receptors desensitization is not observed after treatment with low doses of MAGL inhibitors (Feliszek et al., 2016), and other studies indicate that 2-AG signaling is a critical regulator of emotional behavior, particularly after exposure to stressful stimuli. Also, DAGL-deficient mice or other manipulations that reduce 2-AG signaling in the hippocampus increased behavioral despair and reduced hippocampal neurogenesis in mice (Jenniches et al., 2016).

In the case of AEA, genetic or pharmacological inhibition of FAAH seems to produce antidepressant-like effects (Hill et al., 2009; Bambico et al., 2010). A single injection of a FAAH inhibitor decreased passive behavioral coping responses to acute inescapable stress but failed in producing an antidepressant effect in chronically stressed mice (Xu et al., 2017). Additionally, in WYK rats, repeated treatment with a FAAH inhibitor induced antidepressant-like effect and increased levels of BDNF and AEA in the hippocampus (Vinod et al., 2012). Furthermore, manipulations of the CB system (FAAH KO mice and prolonged treatment with THC) increased the firing rate of dorsal raphe nucleus neurons and enhanced hippocampal 5-HT_1A_ receptor activity, all hallmarks of classical antidepressant mechanism of action (Bambico et al., 2010, 2012). A recent study from the same group found that repeated treatment with a FAAH inhibitor or citalopram also modified the functional states of 5-HT1_A_ and 5-HT2_A/C_/receptors in the hippocampus (Bambico et al., 2016).

Finally, it has been suggested that the available treatments for major depressive disorder modulate cannabinoid signaling (Bambico et al., 2009; Gorzalka and Hill, 2009). Similar to the actions of conventional antidepressants, both exogenous cannabinoids and eCBs seem to regulate serotonergic and noradrenergic systems. Muntoni et al. (2006) have shown that the acute intravenous injection of WIN55212-2 or Δ9-THC increased the firing rate of noradrenergic neurons in the locus coeruleus, while a single administration of the CB_1_ antagonist SR141716A had the opposite effect. Moreover, the inhibition of anandamide hydrolysis by URB597 in rats submitted to an acute swim stress procedure resulted in increased release of noradrenaline in the prefrontal cortex and the basolateral amygdala (Bedse et al., 2015). Gobbi et al. (2005) described that in rats the antidepressant-like activity of acute and chronic URB597 treatment were accompanied by an increase in the firing rate of serotonergic neurons in the dorsal raphe nucleus. In mice lacking the FAAH enzyme, an increased firing rate of serotonergic neurons accompanied by desensitization of inhibitory serotonergic receptors in the hippocampus was reported (Bambico et al., 2010). Similar results were reported by Bambico et al. (2016), that also described a desensitized state of 5-HT2A/C and 5-HT1A receptors in the hippocampus following URB597 treatment. An interrelationship between conventional antidepressants and cannabinoids also come from the similarity of their neuroplastic effects, with both affecting synaptic plasticity, neurogenesis and neurotrophin expression in the hippocampus (Aguado et al., 2005; Jiang et al., 2005; Campos et al., 2015; Zhang et al., 2015).

## eCBs and Hippocampal-Dependent Learning and Memory Task

Emotional learning is essential for survival. Stress and arousal events activate neurobiological systems that play a crucial role in memory consolidation, ensuring that the strength of memories occurs according to their emotional significance (McGaugh, 2015). Cannabinoids can modulate the different phases of memory processes, namely acquisition, consolidation, retrieval, reconsolidation and extinction (Da and Takahashi, 2002; de Oliveira Alvares et al., 2008a, b; Wise et al., 2009; Abush and Akirav, 2010; Santana et al., 2016). For instance, THC impaired the performance of rodents in a working memory task and the acquisition of spatial learning in the water maze, whereas consolidation and retrieval of a previously learned task were not affected. Pre-treatment with a CB_1_ receptor antagonist prevented these learning deficits (Da and Takahashi, 2002). Intrahippocampal injection of a CB_1_ antagonist completely attenuated the memory disruption effect of THC (Wise et al., 2009). Additionally, THC activation the CB_1_ receptors (found mainly in GABAergic interneurons) stimulated the mTOR pathway in the hippocampus through a glutamatergic mechanism, which could be responsible for the characteristic long-term memory impairment induced by cannabinoids (Puighermanal et al., 2009).

Similar to the anxiety data, it seems that the degree of aversiveness is a crucial contributor to the influence of cannabinoid signaling on memory processes (for review, see Morena and Campolongo, 2014). Regarding memory acquisition, systemic injection of an AEA transport inhibitor (AM404) modified cognitive parameters depending on the level of emotional arousal. In a high aversive condition the drug impaired the memory acquisition of the novel object in a recognition task while, in a lower stressful environment, AM404 did not reduce memory performance (Campolongo et al., 2012). Additionally, acute administration of WIN55, 212-2 disrupted the acquisition of contextual-fear conditioning, which is a hippocampal-dependent learning and memory task (Pamplona et al., 2006). On the other hand, regarding memory consolidation, Morena et al. (2014) demonstrated that infusion of a FAAH inhibitor (URB597) into the amygdala, mPFC or hippocampus enhanced memory consolidation only in rodents trained under a high arousal condition. This effect was prevented by a CB_1_ antagonist. Indeed, intrahippocampal infusion of a CB_1_ receptor antagonist disrupted long-term memory consolidation, possibly by inhibiting LTP (de Oliveira Alvares et al., 2005, 2006).

The conflicting findings regarding the influence of cannabinoids in memory modulation could be reflecting differences on the aversiveness of the experimental conditions or the memory phase being tested (Barros et al., 2004; Pamplona et al., 2006; de Oliveira Alvares et al., 2008a, 2010; Abush and Akirav, 2010; Lin et al., 2011; Campolongo et al., 2012). For example, a CB_1_ antagonist injected into the hippocampus, for example, promoted contextual fear memory formation (Lin et al., 2011), impaired the aversive memory extinction process (de Oliveira Alvares et al., 2008b; Abush and Akirav, 2010), and potentiated the reconsolidation of fear memory (de Oliveira Alvares et al., 2008b). On the other hand, facilitation of CB_1_ receptor signaling in this brain area impaired contextual fear memory formation (Lin et al., 2011), facilitated the extinction learning process (de Oliveira Alvares et al., 2008b; Abush and Akirav, 2010), and disrupted emotional memories reconsolidation (de Oliveira Alvares et al., 2008b; Santana et al., 2016). Recently, Micale et al. (2017) also verified that the eCB uptake inhibitor AM404 facilitated safety learning through activity propagation to CA_1_ in a CB_1_-dependent manner, indicating a crucial involvement of the dorsal hippocampus in this process.

## Conclusion

The data revised here indicate that the hippocampal eCB signaling contributes to emotional and behavioral flexibility during the exposure to aversive stimuli, functioning as a regulatory buffer system for emotional responses. eCBs are essential players in plastic events involved in the flexibility of hippocampal functions in basal conditions and during stressful situations. They also regulate the HPA axis responses to stress (Figure 2). Impairment of eCB signaling in the hippocampus following acute/chronic stress could contribute to the development of psychiatric disorders such as major depressive and anxiety disorders (Gorzalka et al., 2008; Lutz, 2009). Finding new eCBs molecular targets to modulate the “stressed hippocampus”, therefore, could be a helpful contribution for novel therapeutic approaches. This proposal, however, has its limitations. Both cannabinoids and stress induce bell- or U-shaped dose/intensity responses, which somehow complicate data interpretation and could help to explain some of the contradictory results reported in the literature. New systematic studies, isolating and comparing in standard conditions the behavioral and neuroplastic effects of mild/intense stressors, acute/repeated stress exposure and treatments, and low/high cannabinoid doses, are needed to elucidate the precise role of the eCBs in stress-induced brain changes.

Another critical point is that most of the available data in the literature associating hippocampus, eCB, and stress arrive from preclinical studies and sometimes seem to depend on the animal model being used. Although stressful experiences are definite risk factors for the precipitation of psychiatric disorders, caution must be taken to translate the preclinical data directly into human pathology. Moreover, besides the hippocampus, other brain regions such as the prefrontal cortex, amygdala (Hill et al., 2010), nucleus accumbens (Bosch-Bouju et al., 2016), the paraventricular nucleus of the hypothalamus (Wamsteeker et al., 2010), lateral habenula (Park et al., 2017) and the periaqueductal gray matter (Moreira et al., 2009), are involved in stress-induced behavioral and neuroplastic consequences. These regions are modulated by eCBs and, therefore, are also potential therapeutic targets for cannabinoid drugs (For additional information about this topic, please read the excellent reviews by Gorzalka and Hill (2009), Riebe and Wotjak (2011), Hillard et al. (2016), Lutz et al. (2016) and Balsevich et al. (2017).

## Author Contributions

FFS, CV-V, NCF-J and VLD wrote the first version of the manuscript. FFS and CV-V edited the first version of the text. ACC and FSG corrected and edited the final version of the manuscript. ACC and NCF-J designed the figures.

## Conflict of Interest Statement

Author NCF-J has a fellowship with the Fundação de Apoio ao Ensino, Pesquisa e Assistência do Hospial das Clínicas da Faculdade de Medicina de Ribeirão Preto e Prati-Donaduzi (Toledo-Brazil). Author FSG is a co-inventor (Mechoulam R, JC, Guimãraes FS, AZ, JH, Breuer A) of the patent “Fluorinated CBD compounds, compositions and uses thereof. Pub. No. WO/2014/108899. International Application No. PCT/IL2014/050023” Def. US no. Reg. 62193296; 29/07/2015; INPI on 19/08/2015 (BR1120150164927). The other authors declare that the research was conducted in the absence of any commercial or financial relationships that could be construed as a potential conflict of interest.
